# An experimental research in mice on the “soft tissue reaction to 3 different mesh implants: Titanium silk, Parietene Progrip and Prolene”

**DOI:** 10.1016/j.jpra.2018.07.005

**Published:** 2018-08-03

**Authors:** Viktor Eduardovich Kobazev, Manish Kumar Yadav, Andrey Vyacheslavovich Vasilyev, Alexander Ivanovich Nerobeev

**Affiliations:** a Central Research Institute of Dental and Maxillofacial surgery (CRID), Moscow, Russian Federation; b Peoples' Friendship University of Russia, Moscow, Russian Federation; c Department of cosmetology, Maxillofacial and plastic surgery, Russian Medical Academy for Continuing Postgraduate Education (RMACPE), Moscow, Russian Federation

**Keywords:** Titanium silk, Parietene Progrip, Prolene, Mesh implant, Histological mesh analysis, Static facial nerve repair

## Abstract

**Purpose:**

To analyze the soft tissue reaction of ‘Titanium Silk’ mesh implant in comparison with ‘Parietene Progrip’ and ‘Prolene’ mesh implants for the reinforcement and augmentation of soft tissues to improve the results of static correction in Facial Paralysis and other defects of Maxillofacial region.

**Materials and methods:**

Under standard laboratory conditions, 89 mice were divided into 4 groups: a control group of 5 mice; first group of 28 mice with Titanium mesh implant, second group of 28 mice with semi-resorbable ‘Parietene Progrip’ implant and third group of 28 mice with ‘Prolene’ implant. Under inhalational anesthesia with ethyl ether at days 7, 14, 30 and 60, seven mice from each experimental group underwent Gross and histological analysis of the mesh structures for the following characteristics: Macrophage Infiltration, Multinucleated Macrophages, Meshwork around the implant fibers, Connective tissue proliferation, Angiogenesis and Fibroblasts.

**Results:**

Histological analysis revealed a significantly less pronounced inflammatory response to Titanium mesh implant resulting in the formation of a more delicate connective tissue network around the mesh elements.

**Conclusion:**

The experiment clearly demonstrated the cellular and tissue responses to different implantable mesh materials at various times of its integration. It revealed that the titanium mesh is the most bio-inert alloplastic material suitable for reinforcement of soft tissue augmentation and to prioritize it's use in static correction of facial paralysis and other defects of the maxillofacial region. A postoperative timeframe of 30 days is considered appropriate for the adequate formation of connective tissue around the mesh elements.

## Graphical abstract

**Figure:**
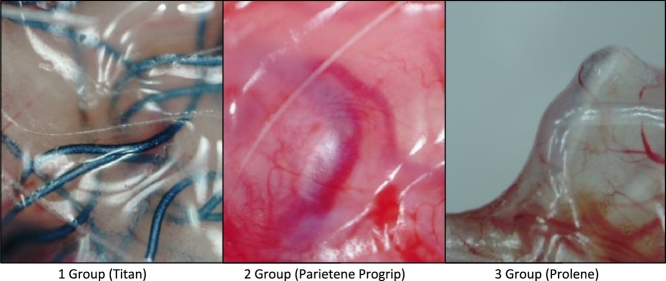

**Macrophotographs (x200) 60**th **day:** On the 60th day of the experiment, 7 mice were executed from each group (*n* = 21). Soft tissue edema in the implant area is not seen. In the 1st group, formation of a soft connective tissue capsule loosely adherent to the adjacent connective tissue structures (dermis, superficial fascia) around the implant is seen. The reinforcement of the vascular pattern in the implant zone in the 2nd and 3rd experimental group is preserved. The connective tissue around the implants is well formed. Formation of coarse scar tissue is not noted. The connective tissue "covering" around the implants is strongly adherent to the superficial fascia and skin. The scarring process is more pronounced in the 3rd group. Suppuration was not seen.

## Introduction

The reconstructive challenge posed by complete facial paralysis is to optimally restore meaningful facial function and an acceptable cosmesis with minimal residual patient morbidity^1^.

Although meshes are the most commonly used biomaterials in medical practice, with approximately 1.5 million implants used per year^2^, numerous questions remain unaddressed about the host inflammatory response induced by mesh implants^3^.

The utilization of alloplastic material (i.e. silicone prostheses, polypropylene mesh, etc.) in plastic surgery has been widely accepted. Polypropylene (PP) is the most commonly used material to manufacture meshes, nonetheless several other absorbable and non-absorbable materials are also being used^4^, ^5^.

Whatever be the nature of the material employed, some inflammatory reaction is bound to occur^6^.

The host response to implanted mesh follows a cascade of events involved in wound healing including coagulation, inflammation, angiogenesis, epithelialization, fibroplasia, matrix deposition, and contraction^7^, ^8^, ^9^.

At the same time, experimental data reveal that material composition and mesh structure may significantly affect foreign body reaction.^10^

Mesh characteristics such as pore size, chemical composition, filament structure, amount of implanted material, and biodegradability affect the processes of inflammation, angiogenesis, and tissue formation which consequently may alter wound healing^11^, ^12^, ^13^, ^14^, ^15^, ^16^, ^17^. Theoretically, the increased diameter of the pores and the reduction in the density of meshes could minimize inflammation and, consequently reduce the complications related to these implants^18^, ^19^, ^20^, ^21^.

According to data from current randomized controlled trials and retrospective studies, light meshes seem to have some advantages with respect to postoperative pain and foreign body sensation. Experimental studies have shown that the inflammatory response of an organism toward titanium-coated meshes is much reduced when compared with other implants.^21^, ^22^

Bearing this in mind, this experiment was performed to test the reaction of soft tissues to Titanium mesh implant (“Titanium Silk” developed by TsKB RAS, manufacturer: OOO TEMP, Yekaterinburg, Russia).

**Table:** 

Properties of “Titanium silk”:	
Features	Value
Composition	99.9% titanium
Surface density (Weight)	35–60 g/m ^2^
Pore size	1–3 mm
Thread thickness (Filament diameter)	65 μm (65 μm)
Porosity (3D–weaving)	91%
Elasticity (Physiological elasticity at 16 N/cm)	38–46%
(More details can be found at the Manufacturer's website: http://titanell.com/2015/05/15/another-interesting-single-post/)	

This article is the outcome of an elaborate research in mice on tissue response to Titanium mesh implant with subsequent clinical application for soft tissue reinforcement.

## Objective

The objective of this experiment has been to determine the least reactive and highly efficient alloplastic material for mesh implant in treating Facial Paralysis and mandibular injuries revealing minimal inflammatory response along with reduced postoperative pain and foreign body sensation.

## Materials and methods

The experiment was conducted under standard laboratory conditions. A group of 89 mice was randomly segregated into four groups with a control group of 5 mice serving as a comparative evaluation of general health condition and behavioral reactions as implantation was not performed in this group. The first experimental group of 28 mice was implanted with a titanium mesh implant “Titanium Silk”. In the second experimental group of 28 mice, a self-fixating partially bio-degradable mesh implant based on polylactic acid and polypropylene “Parietene Progrip” was implanted while in the third group of 28 mice, a polypropylene mesh implant “Prolene” was implanted.

All experimental operations were carried out under inhalation anesthesia with ethyl ether. At the withers, following antiseptic treatment, a sharp linear incision of 15 mm was made. Further, blunt dissection was done to create a subcutaneous pocket of 15 × 15 mm and a sterile implant sample of 10 × 10 mm was placed. Skin was closed with sutures and antiseptics applied. The stages of implantation are shown in Video 1 - Stages of the experimental operation.

**Figure 1:**
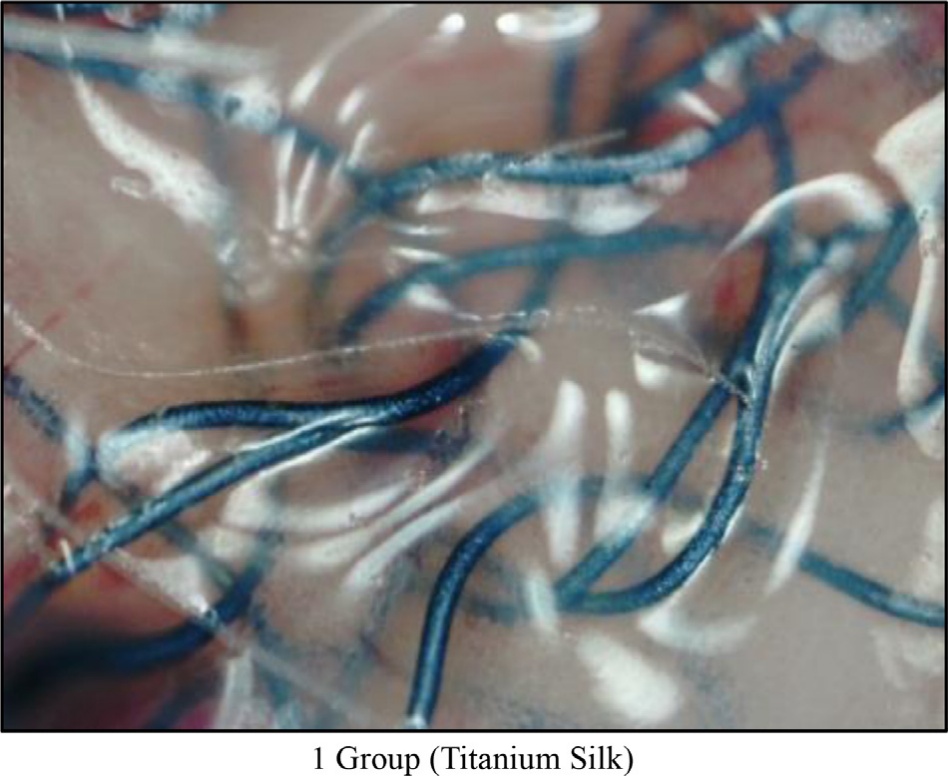
Gr 1 Macro-Photo `Titanium Silk' (Day 60).

In each group, the overall health condition of the mice, including behavioral responses and body mass dynamics were evaluated.

Clinically, in the wound area, postoperative edema was observed in all groups for the first 2 days. There was no marked painful reaction during palpation over the implant placement area. Subsequently, all wounds healed with primary intention, without any suppuration.

Following euthanasia with 100% CO_2_, musculocutaneous tissue units of 7 mice (from each experimental group with sample implants) were studied on days 7, 14, 30, and 60. A macro preparation was made and macrophotography was carried out using a digital USB microscope MIKMED-LCD (PRC) with magnification of 100–200 times (Figures 1, 2 and 3). Also, the resulting tissue samples were fixed with 10% formalin solution and micro preparations were made for histological examination.

The software ‘GraphPad Prism 7 (USA)’ was used for statistical evaluation of data and creation of graphs. For sorting of groups, the different types of implant were chosen.

As the groups included a small number of observations (*n*=7), and the values of investigated parameters were presented in a point discrete scale, bilateral non-parametric tests were used to identify inter- and intra-group differences.

Comparison between the three study groups, at the same observation times, were performed using the Kruskal–Wallis test with Dunn's *post hoc* test. The dynamics of changes in the study parameters during the observation period was also assessed using Dunn's test for independent samples.

## Results

### General observation

In the experimental groups, there were no clinically significant differences among the mice during the postoperative period. The animals were active within an hour of operation. During observation, typical behavioral reactions known for these types of experimental animals were noted in all groups: mice were actively moving around the cell, food and water was consumed normally. The daily remainder of food and drink in animals in the groups did not differ significantly. The increase in body weight, as an integral measure of the general state of the animal, is presented in Table 1.

**Table 1:** Weight gain of experimental animals.

Group	The periods of observation, weight (gms)				
	Initially	7 days	14 days	30 days	60 days
Control	287 ± 28	344 ± 31	361 ± 28	364 ± 20	409 ± 29
1- Group	291 ± 46	306 ± 38*	345 ± 48	329 ± 43	396 ± 39
2- Group	273 ± 49	303 ± 48*	321 ± 42*	296 ± 54*	383 ± 45
3- Group	283 ± 38	306 ± 38*	333 ± 40*	340 ± 47	407 ± 26

⁎ statistically significant difference (*p* ≤ 0.05) to the control group in the same period of observation (to evaluate the statistical significance of differences between groups ‘Mann–Whitney *U* test’ was used)

### Gross anatomy

Refer to Macro-Photographs at Day 60:

1. The results of an indirect assessment of bio-compatibility of implant studies showed that titanium-containing reticular implant “TITANIUM SILK” was the most bio-inert. This group of mice quickly gained weight, and formed a soft connective tissue capsule loosely adherent to the adjacent connective tissue structures (dermis, superficial fascia) around the implant (Figure 1).
2. Self-fixating semi-resorbable mesh implant “PARIETENE PROGRIP” caused marked neo-angiogenesis inducing a tissue reaction. A firmly adherent connective tissue capsule was formed, which led the implant to be tightly fixed to the dermis and superficial fascia (Figure 2).

**Figure 2:**
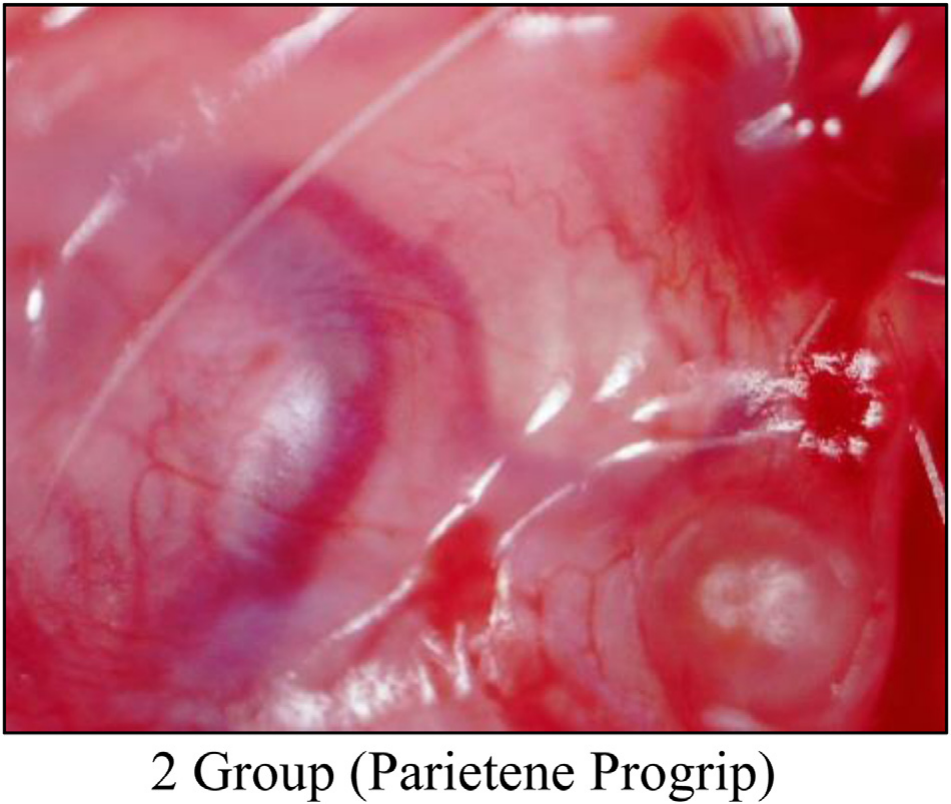
Gr 2 Macro-Photo 'Parietene Progrip' (Day 60).
3. Implants “PROLENE” were characterized by the formation of a pronounced connective tissue capsule, which tended to be tightly fixed to the superficial fascia but had loosely and sparsely adherent to the dermis (Figure 3).

**Figure 3:**
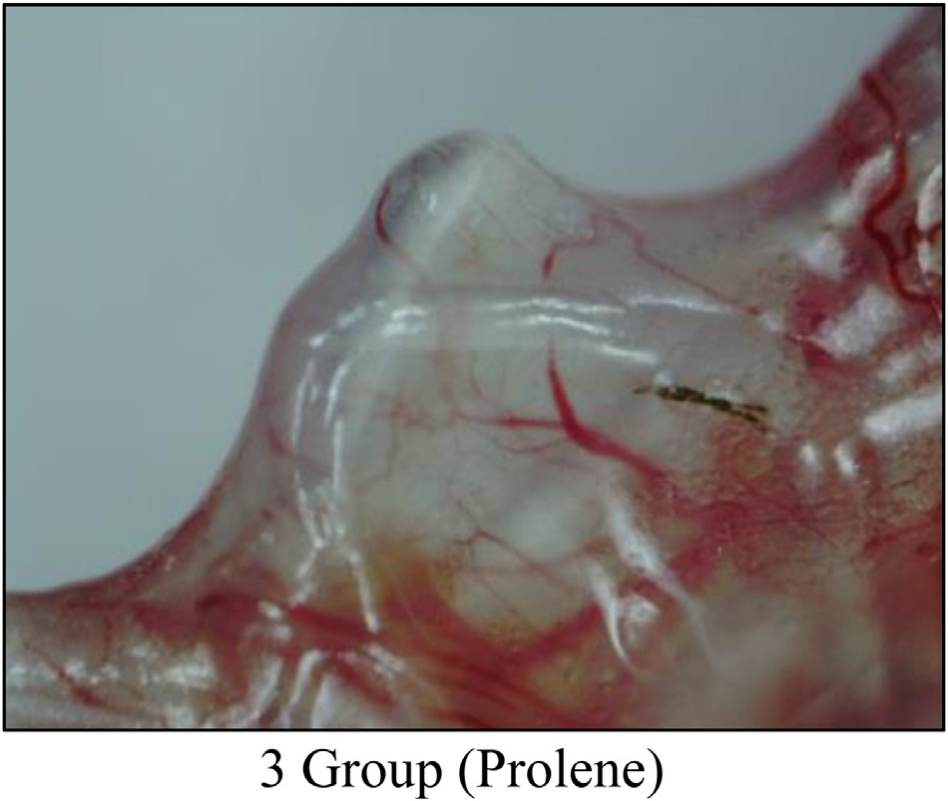
Gr 3 Macro-Photo 'Prolene' (Day 60).

The final conclusions about the nature of tissue reaction to the implants under investigation can be formulated according to the results of its histological examination.

### Histological analysis

#### Macrophage infiltration

**Figure 4:**
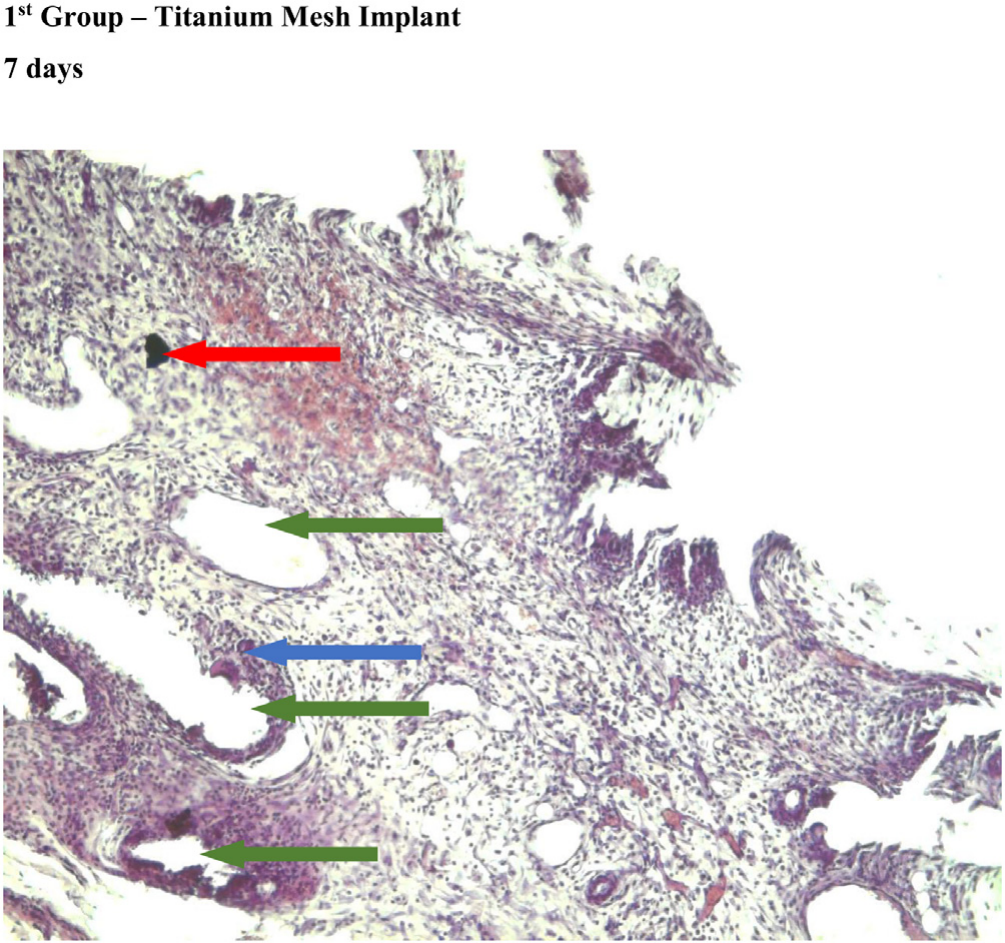
Gr 1-Titan 7 days.

**Graph 1:**
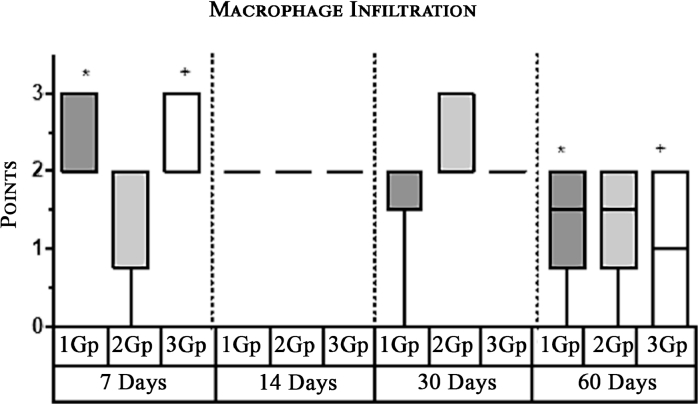
Intensity of macrophage infiltration in the comparison group at different days. **Herein and hereafter:** The symbol "–––" indicates a statistically significant **Inter-group difference** (Dunn's test, *p*<0.05). The symbols *, **, ***, +, # etc., indicates **Intra-group differences** between the study parameters at different time periods, identified in pairwise comparison (Dunn's test, *p*<0.05).

In the first and third group, which used titanium mesh and polypropylene mesh implant "Prolene" respectively, a statistically significant greater macrophage infiltration was observed on the 7th day as compared to the 60th day (Figures 4,Figure 7, Figure 12, Figure 14, Graph 1, Table 2, 3). In the second group with “Parietene Progrip”, it was less expressed on the 7th day, increasing on the 14th day with a marked decrease on the 30^th^ day (Figure 8, Figure 9, Figure 10, Graph). Statistically significant differences between the study groups throughout the periods of observation were not detected (Table 4).

Scoring points for ‘Macrophage infiltration’:
1 point - single macrophages;
2 points - moderate macrophage infiltration;
3 points - pronounced macrophage infiltration.

**Table 2:** Values in points amidst the groups over time.

Group	Histological analysis	7 Days	14 Days	30 Days	60 Days
**FIRST group**	Macrophages Infiltration	2 [2; 3]	2 [2; 2]	2 [1.5; 2]	1.5 [0.75; 2]
	Giant Cell Infiltration	1 [0; 1]	0 [0; 1]	0 [0; 0]	0 [0; 1]
	Meshwork around Fibers	0 [0; 0]	0 [0; 1]	0 [0; 0]	0 [0; 0]
	Connective Tissue Volume	3 [3; 3]	2 [2; 2]	2 [2; 2]	2 [2; 2]
	Vascular bed volume	1 [1; 2]	2 [1; 2]	1 [0; 1]	1 [1; 2]
	Fibroblast density	2 [2; 2]	1 [1; 1]	1 [1; 2]	1 [1; 1]
**SECOND group**	Macrophages infiltration	2 [0.75;2]	2 [2;2]	2 [2;3]	1.5 [0.75;2]
	Giant cell infiltration	1 [0;2]	0 [0;1]	0 [0;2]	1 [0;1]
	Meshwork around fibers	0 [0; 0]	0 [0; 0]	0 [0; 1]	1 [1; 1]
	Connective tissue volume	3 [2; 3]	3 [3; 3]	3 [2; 3]	2 [2; 2]
	Vascular bed volume	1 [1; 2]	1 [1; 2]	1 [1; 2]	2 [2; 2]
	Fibroblast density	1 [1; 2]	1 [1; 2]	2 [1; 2]	1 [1; 1]
**THIRD group**	Macrophages infiltration	2 [2;3]	2 [2;2]	2 [2;2]	1 [0;2]
	Giant cell infiltration	1 [1;1]	1 [0;1]	1 [1;1]	0 [0;1]
	Meshwork around fibers	0 [0; 1]	1 [0; 1]	0 [0; 1]	1 [1; 1]
	Connective tissue volume	2 [2; 3]	2 [2; 3]	2 [2; 2]	2 [2; 2]
	Vascular bed volume	2 [1; 2]	2 [2; 2]	1 [1; 2]	2 [1; 2]
	Fibroblast density	2 [1; 2]	1 [1; 2]	1 [1; 2]	1 [1; 1]

**Table 3:** INTRA-group changes over time (Dunn test).

Group	Histological analysis		At 7 & 14 days	At 7 & 30 days	At 7 & 60 Days	At 14 & 30 Days	At 14 & 60 Days	At 30 & 60 Days
**FIRST Group**	Macrophages infiltration	*P*	>0.99	0.51	0.03	>0.99	0.51	>0.99
		Difference?	No	No	**Yes**	No	No	No
	Giant cell infiltration	*P*	>0.99	0.04	0.6	0.6	>0.99	>0.99
		Difference?	No	**Yes**	No	No	No	No
	Meshwork around fibers	*P*	0.25	>0.99	>0.99	0.25	0.25	>0.99
		Difference?	No	No	No	No	No	No
	Connective tissue volume	*P*	0.003	0.02	0.02	>0.99	>0.99	>0.99
		Difference?	**Yes**	**Yes**	**Yes**	No	No	No
	Vascular bed volume	*P*	>0.99	0.12	>0.99	0.03	>0.99	0.12
		Difference?	No	No	No	**Yes**	No	No
	Fibroblast density	*P*	0.003	0.12	0.003	>0.99	>0.99	>0.99
		Difference?	**Yes**	No	**Yes**	No	No	No
**SECOND Group**	Macrophages infiltration	*P*	>0.99	0.21	>0.99	>0.99	0.59	0.05
		Difference?	No	No	No	No	No	No
	Giant cell infiltration	*P*	>0.99	>0.99	>0.99	>0.99	>0.99	>0.99
		Difference?	No	No	No	No	No	No
	Meshwork around fibers	*P*	>0.99	>0.99	0.006	>0.99	<0.001	0.04
		Difference?	No	No	**Yes**	No	**Yes**	**Yes**
	Connective tissue volume	*P*	>0.99	>0.99	0.7	>0.99	0.04	0.13
		Difference?	No	No	No	No	**Yes**	No
	Vascular bed volume	*P*	>0.99	>0.99	0.21	>0.99	0.05	0.21
		Difference?	No	No	No	No	No	No
	Fibroblast density	*P*	>0.99	>0.99	>0.99	>0.99	>0.99	0.74
		Difference?	No	No	No	No	No	No
**THIRD Group**	Macrophages infiltration	*P*	>0.99	>0.99	0.01	>0.99	0.34	0.34
		Difference?	No	No	**Yes**	No	No	No
	Giant cell infiltration	*P*	0.84	>0.99	0.01	>0.99	0.64	0.19
		Difference?	No	No	**Yes**	No	No	No
	Meshwork around fibers	*P*	>0.99	>0.99	0.08	>0.99	>0.99	0.08
		Difference?	No	No	No	No	No	No
	Connective tissue volume	*P*	>0.99	>0.99	0.16	>0.99	0.61	>0.99
		Difference?	No	No	No	No	No	No
	Vascular bed volume	*P*	>0.99	>0.99	>0.99	0.64	>0.99	>0.99
		Difference?	No	No	No	No	No	No
	Fibroblast density	*P*	>0.99	>0.99	0.04	>0.99	0.64	0.64
		Difference?	No	No	**Yes**	No	No	No

**Table 4:** INTER-group changes over time (Dunn test).

Group	Histological analysis	7 Days		14 Days		30 Days		60 Days	
		*P*	Difference	*P*	Difference	*P*	Difference	*P*	Difference
**FIRST Group & SECOND Group**	Macrophages infiltration	0.15	No	>0.99	No	>0.99	No	>0.99	No
	Giant cell infiltration	>0.99	No	0.2	No	0.2	No	0.85	No
	Meshwork around fibers	>0.99	No	0.55	No	0.55	No	<0.001	**Yes**
	Connective tissue volume	0.85	No	0.07	**Yes**	0.07	No	>0.99	No
	Vascular bed volume	>0.99	No	0.05	No	0.05	**Yes**	0.08	No
	Fibroblast density	0.24	No	0.88	No	0.88	No	0.66	No
**FIRST Group & THIRD Group**	Macrophages infiltration	>0.99	No	>0.99	No	>0.99	No	>0.99	No
	Giant cell infiltration	0.51	No	0.02	No	0.02	**Yes**	>0.99	No
	Meshwork around fibers	0.41	No	0.55	No	0.55	No	<0.001	**Yes**
	Connective tissue volume	0.32	No	>0.99	No	>0.99	No	0.7	No
	Vascular bed volume	>0.99	No	0.05	No	0.05	**Yes**	>0.99	No
	Fibroblast density	>0.99	No	>0.99	No	>0.99	No	>0.99	No
**SECOND Group & THIRD Group**	Macrophages Infiltration	0.15	No	>0.99	No	>0.99	No	>0.99	No
	Giant cell infiltration	>0.99	No	>0.99	No	>0.99	No	0.85	No
	Meshwork around fibers	>0.99	No	>0.99	No	>0.99	No	>0.99	No
	Connective tissue volume	>0.99	No	0.07	No	0.07	No	>0.99	No
	Vascular bed volume	>0.99	No	>0.99	No	>0.99	No	0.29	No
	Fibroblast density	>0.99	No	>0.99	No	>0.99	No	>0.99	No

#### Giant cells (Multinucleated macrophages)

**Graph 2:**
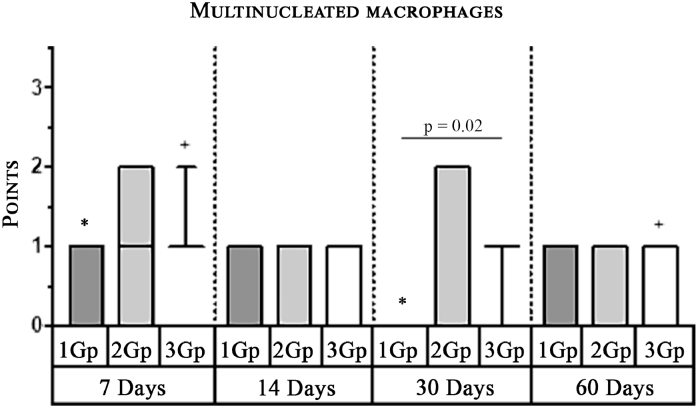
Expression of Multinuclear Macrophages (Giant Cells) infiltration amidst comparison groups over time. **Herein and hereafter:** The symbol "–––" indicates a statistically significant **Inter-group difference** (Dunn's test, *p* < 0.05). The symbols *, **, ***, +, # etc., indicates **Intra-group differences** between the study parameters at different time periods, identified in pairwise comparison (Dunn's test, *p* < 0.05).

In the first group, statistically significant multinucleated macrophage infiltration was observed on the 7th day (Figure 4) in comparison with the 30th day (Figure 6). In the third group, statistically significant large macrophage infiltration was observed on the 7th day (Figure 12) in comparison with that on the 60th day (Figure 14, Graph 2, Table 2, Table 3). Statistically significant differences were noticed on the 30th day of observation: the number of multi-nucleated macrophages in the first group was lower than that in the third group (Table 4).

Scoring points for the presence of ‘multinucleated macrophages’:
0 point - none.
1 point - multinuclear macrophages occur in a single field of vision.
2 points - multinuclear macrophages are found in most fields of vision.

#### Meshwork around the implant fibers

**Graph 3:**
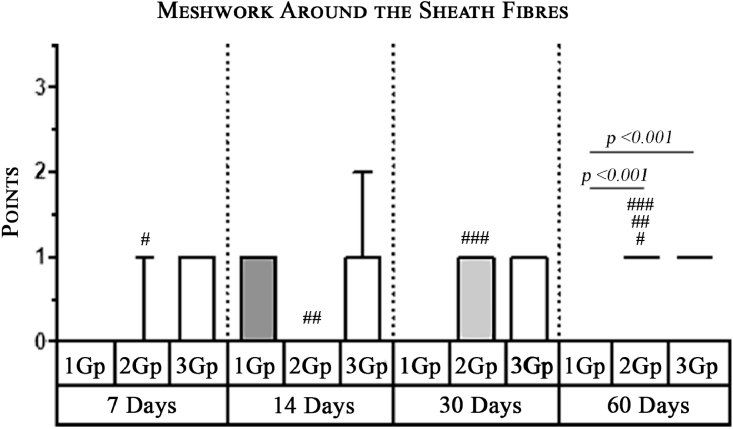
The severity of Meshwork Around the implant fibers between the groups over time. **Herein and hereafter:** The symbol "–––" indicates a statistically significant **Inter-group difference** (Dunn's test, *p* < 0.05). The symbols *, **, ***, +, # etc., indicates **Intra-group differences** between the study parameters at different time periods, identified in pairwise comparison (Dunn's test, *p* < 0.05).

In the second group, where the self-retaining bio-resorbable mesh “Parietene Progrip” was used, a statistically significant increase in the volume of meshwork around the filaments of the mesh was observed on the 60th day (Figure 11) in comparison to all previous periods (Graph 3, Table 2, Table 3). Statistically significant differences between the first and the second, and also between the first and the third group, were found on the 60th day (Figure 7, Figure 11, Figure 14). The volume of meshwork around the filaments in the first group was significantly less than that in the second and the third groups (Table 4).

Scoring points for the presence of connective tissue Meshwork around the implant fibers:
0 point – meshwork is not detected.
1 point - moderately pronounced meshwork.
2 points - significantly pronounced meshwork.

#### Connective tissue

**Figure 5:**
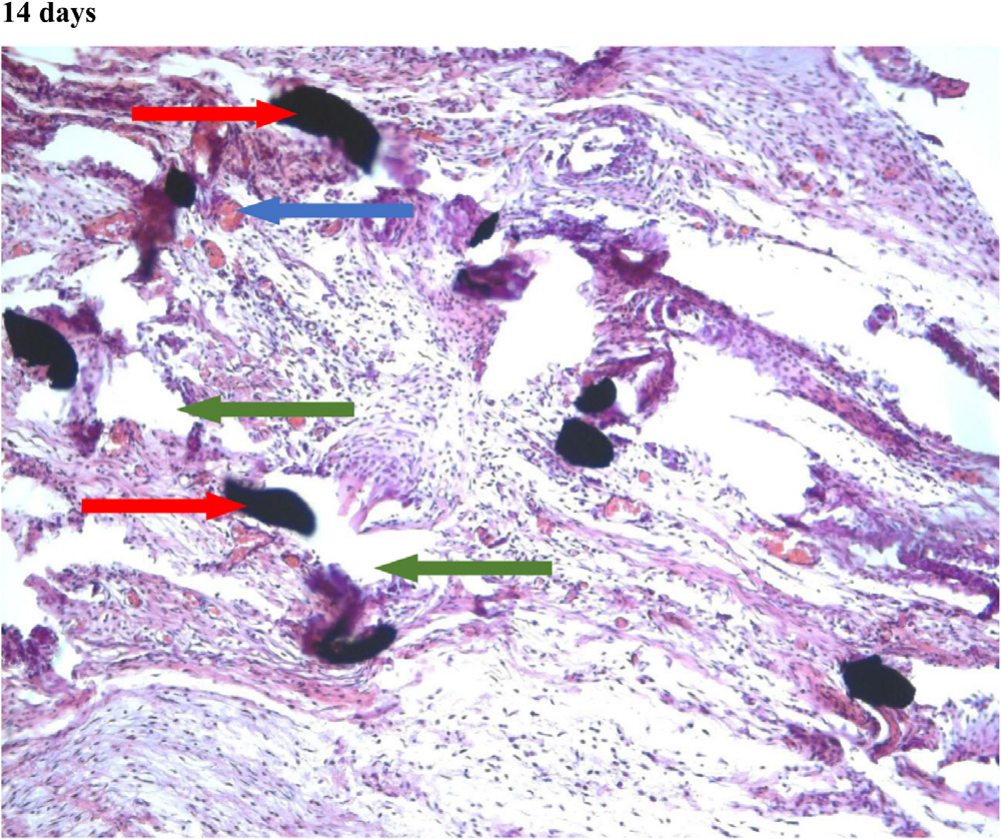
Gr 1-Titan 14 days.

**Figure 6:**
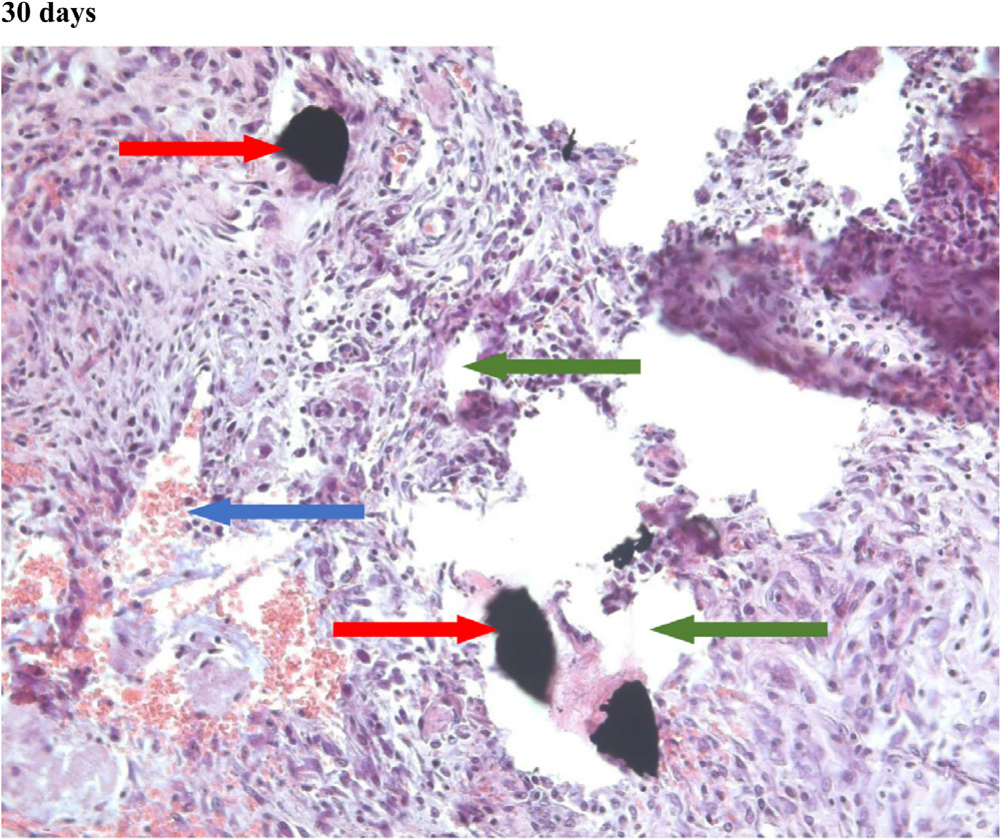
Gr 1-Titan 30 days.

**Figure 7:**
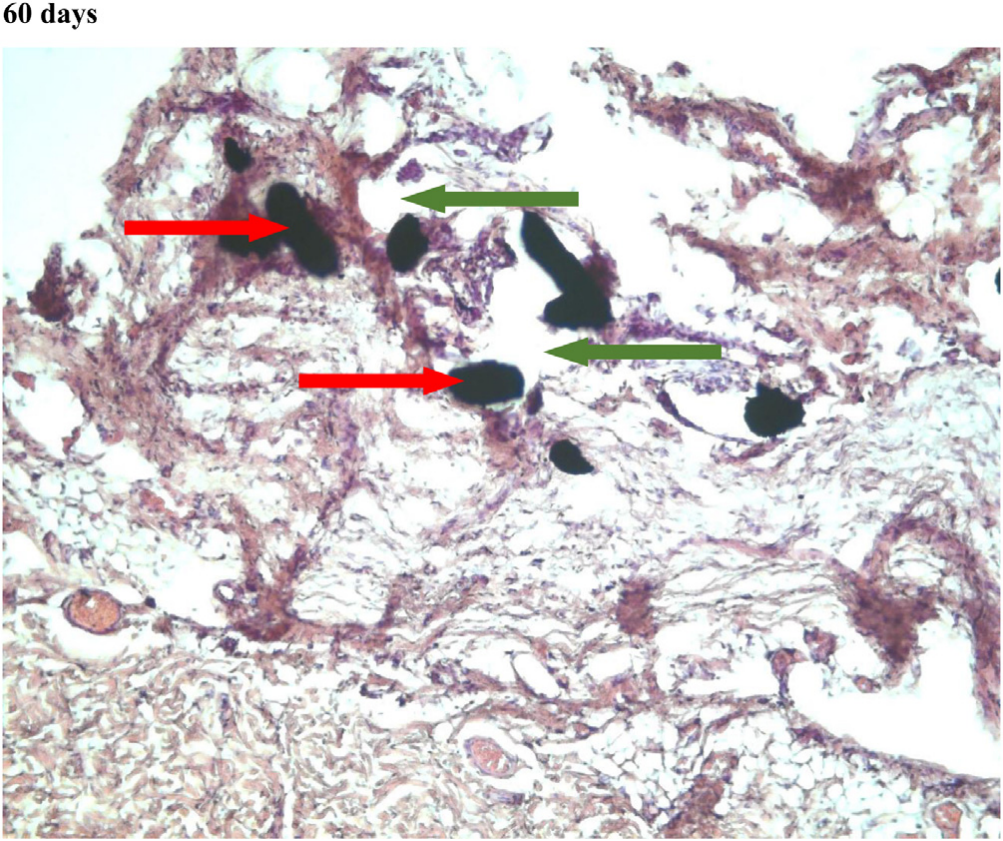
Gr 1-Titan 60 days.

**Graph 4:**
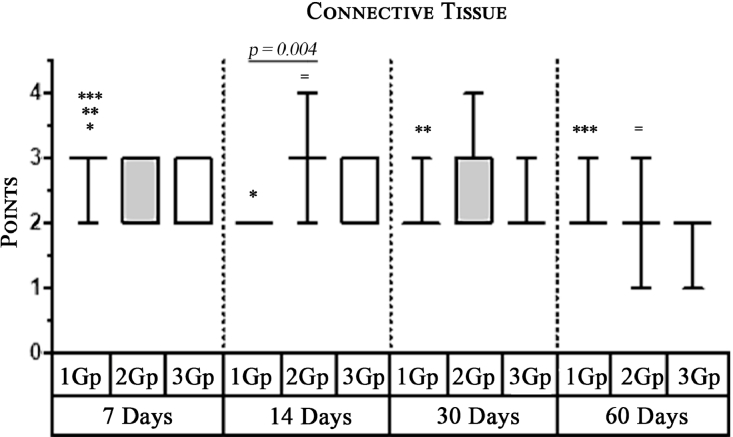
Volume of Connective Tissue between groups over time. **Herein and hereafter:** The symbol "–––" indicates a statistically significant **Inter-group difference** (Dunn's test, *p* < 0.05). The symbols *, **, ***, +, # etc., indicates **Intra-group differences** between the study parameters at different time periods, identified in pairwise comparison (Dunn's test, *p* < 0.05).

In the first group, a significant decrease in the volume of connective tissue was observed on the 14th, 30th and 60th days (Figure 5, Figure 6, Figure 7), compared with that on the 7th day (Figure 4). In the second group, a statistically significant decrease in the volume of the connective tissue on the 60th day (Figure 11) as compared to that on the 14th day (Figure 9, Graph 4, Table 2, Table 3). On the 14th day, a statistically significant less amount of connective tissue was observed in the first group over that in the second (Table 4).

The degree of growth of connective tissue at the beginning and the end of the experiment was approximately the same in all groups. However, there was a characteristic increase of the connective tissue of the second group at the 2nd and 3rd week of experiment.

Scoring points for the formation of connective tissue was assessed by the density of its growth:
1 point - connective tissue not expressed.
2 points - connective tissue weakly expressed.
3 points - connective tissue moderately expressed.
4 points - connective tissue significantly expressed.

#### Angiogenesis

**Graph 5:**
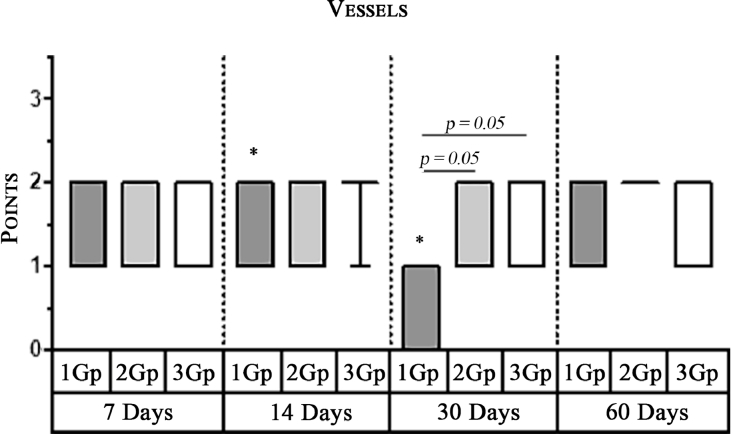
Volume of the Vessels (vascular bed) between groups over time. **Herein and hereafter:** The symbol "–––" indicates a statistically significant **Inter-group difference** (Dunn's test, *p* < 0.05). The symbols *, **, ***, +, # etc., indicates **Intra-group differences** between the study parameters at different time periods, identified in pairwise comparison (Dunn's test, *p* < 0.05).

The first group observed a significant decrease in vascular volume on the 14th day as compared with that on the 7th day (Figure 4, Figure 5, Graph 5, Table 2, Table 3). On 30th day, significantly lesser amount of connective tissue in the first group was observed than that on the second and the third groups (Figure 6, Figure 10, Figure 13, Table 4).

Scoring points for Angiogenesis was as per the number of vessels around the mesh filaments.
0 point - vessels not detected.
1 point - a single vessel seen.
2 points - multiple vessels seen.

#### Fibroblasts

**Figure 8:**
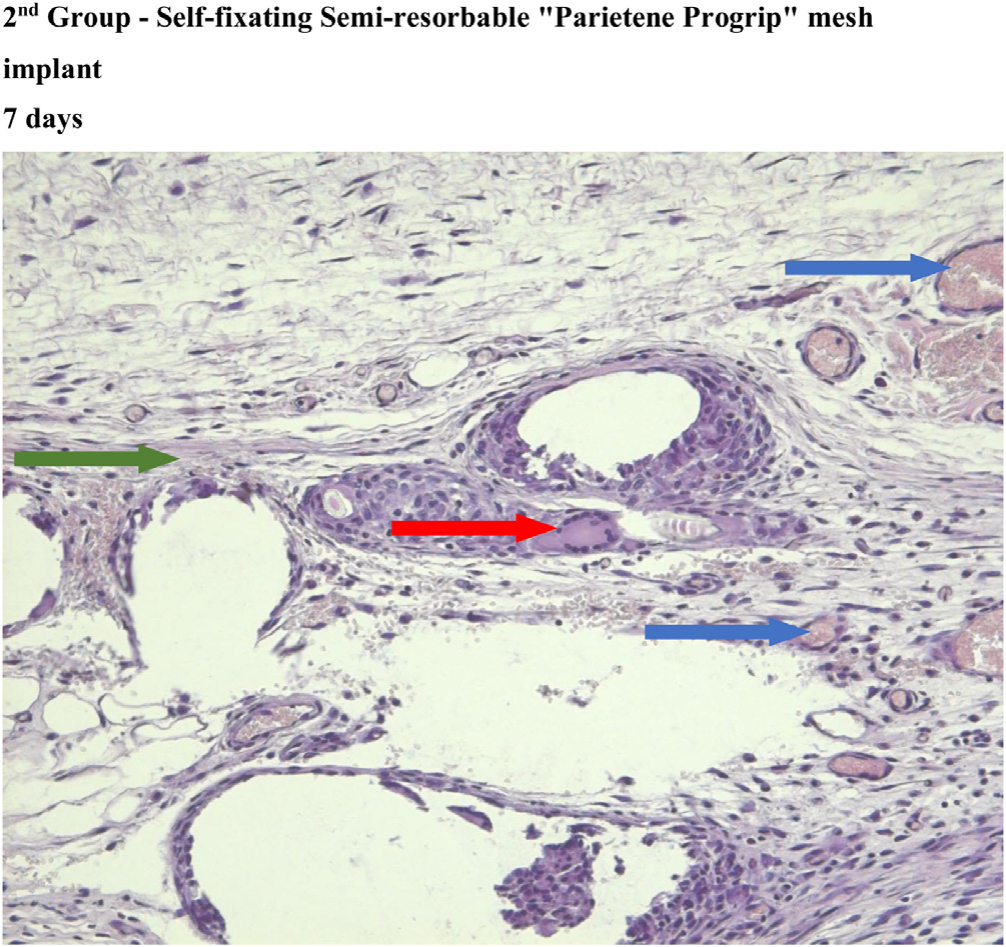
Gr 2-Parietene Pro 7 days.

**Graph 6:**
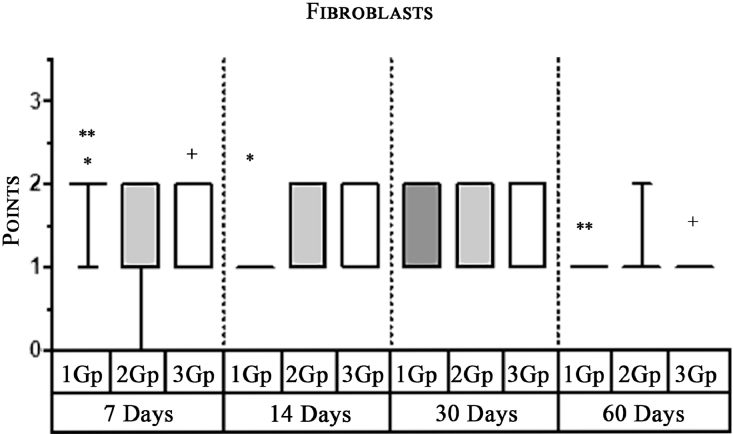
The density of fibroblasts between the groups over time. **Herein and hereafter:** The symbol "–––" indicates a statistically significant **Inter-group difference** (Dunn's test, *p* < 0.05). The symbols *, **, ***, +, # etc., indicates **Intra-group differences** between the study parameters at different time periods, identified in pairwise comparison (Dunn's test, *p* < 0.05).

In the first group, a significantly less number of fibroblasts was observed on the 14th day (Figure 5) and the 60th day (Figure 7) as compared with that on the 7th day (Figure 4). In the second group, a significant decrease was noted on the 60th day (Figure 11) as compared with that on the 7th day (Figure 8, Graph 6, Table 2, Table 3).

Scoring points for the density of fibroblasts:
0 point - fibroblasts are not detected.
1 point – fibroblasts are sparse.
2 points - fibroblasts are densely localized.

**Figure 9:**
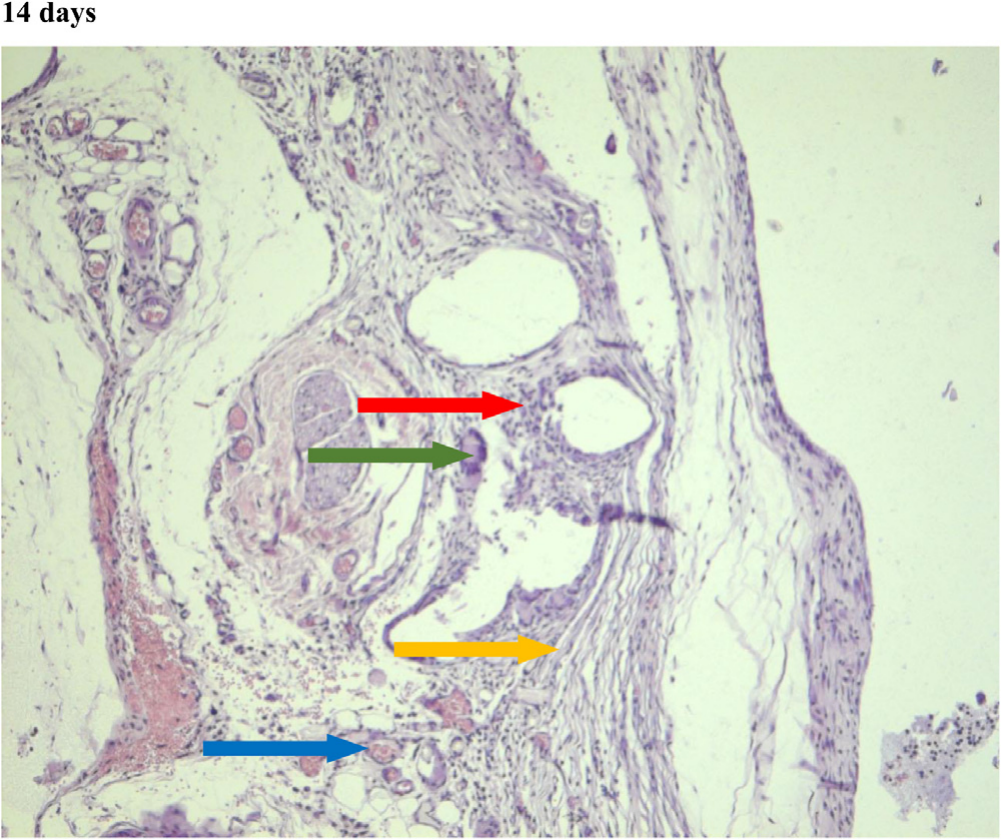
Gr 2-Parietene Pro 14 days.

**Figure 10:**
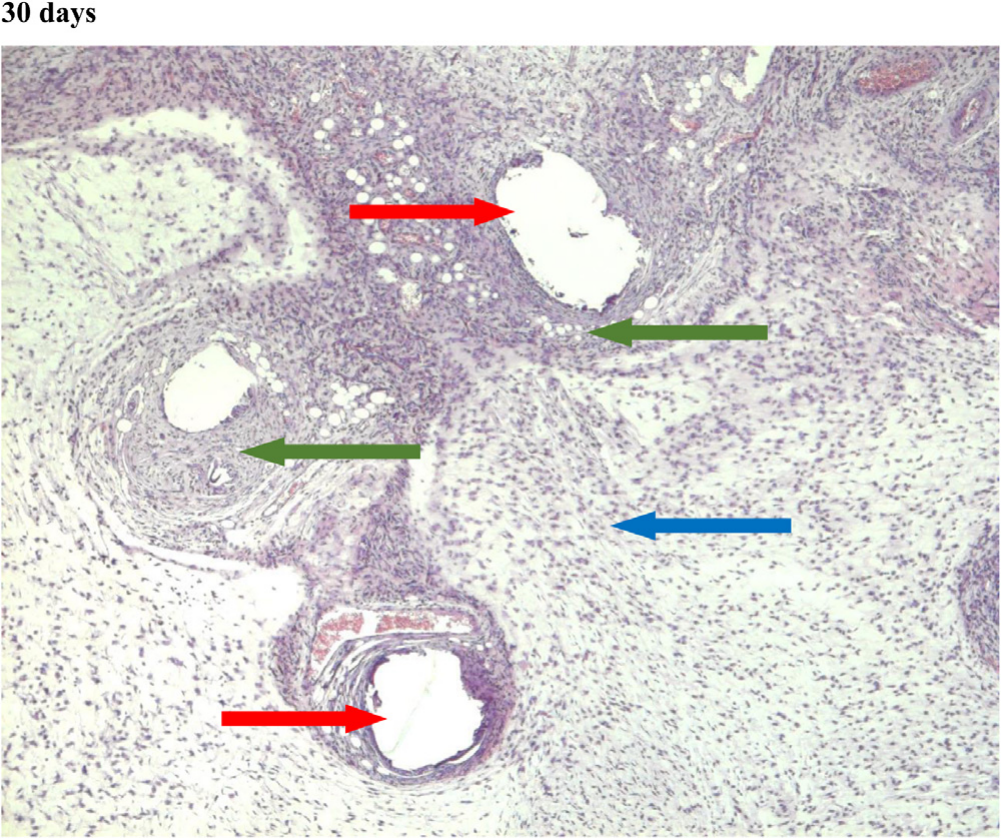
Gr 2-Parietene Pro 30 days.

**Figure 11:**
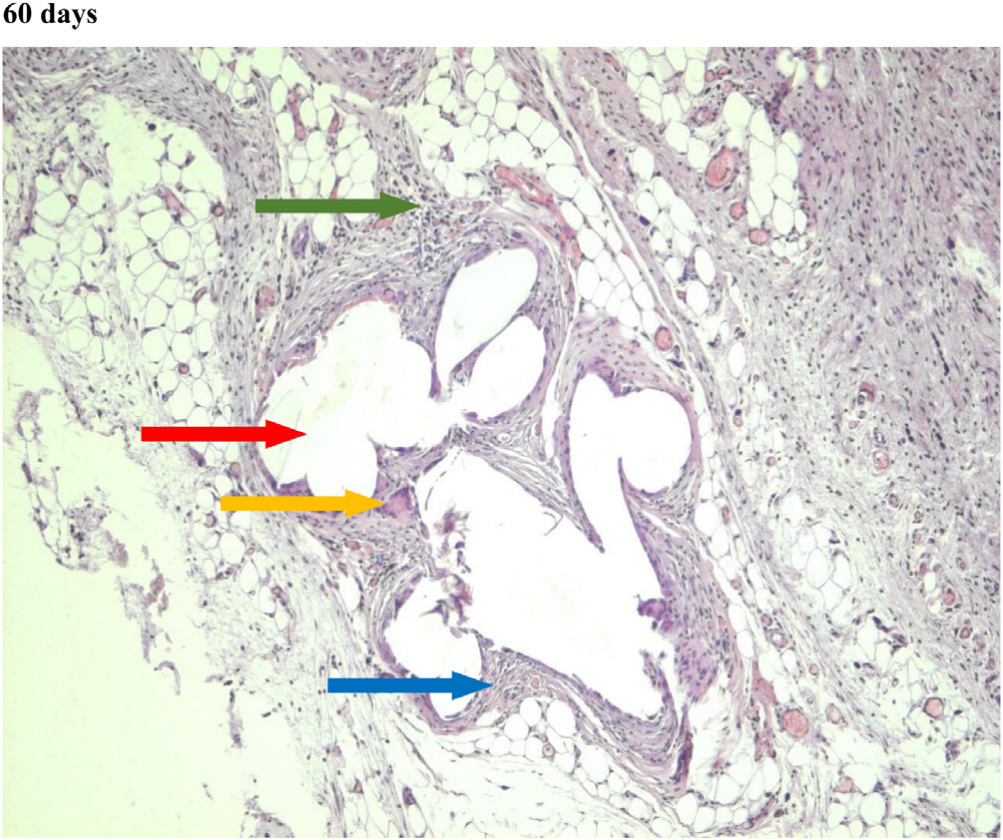
Gr 2-Parietene Pro 60 days.

**Figure 12:**
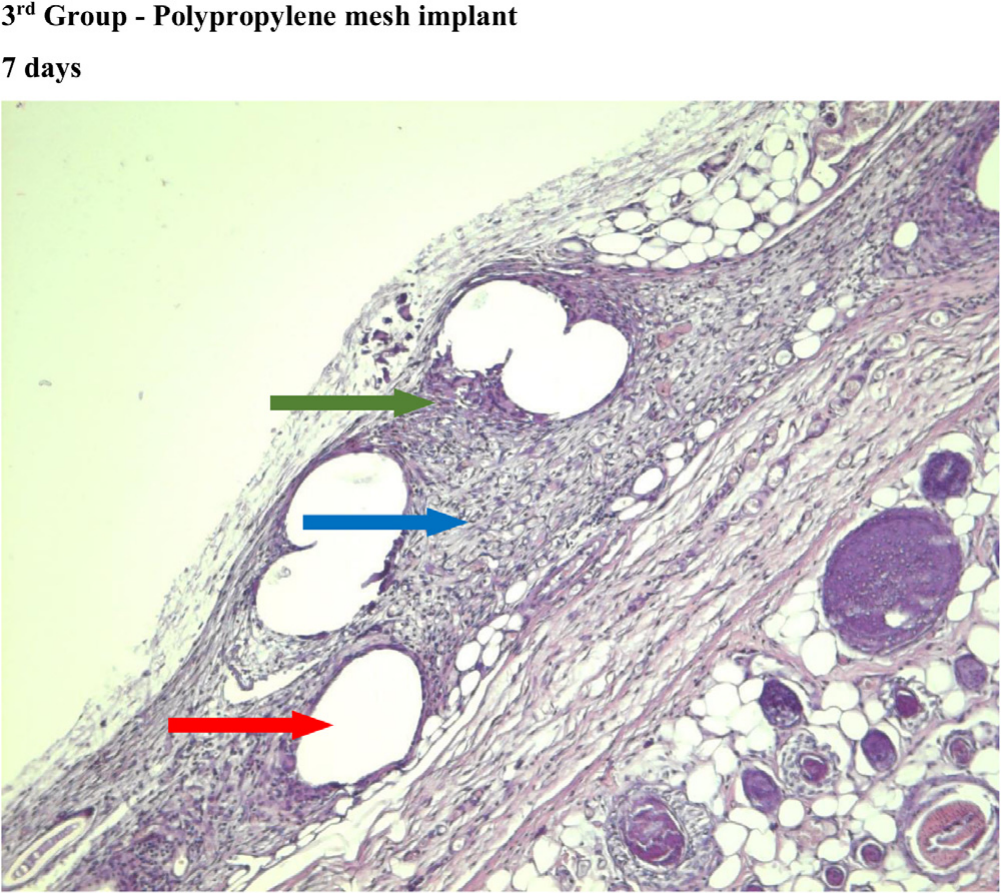
Gr 3-Prolene 7 days.

**Figure 13:**
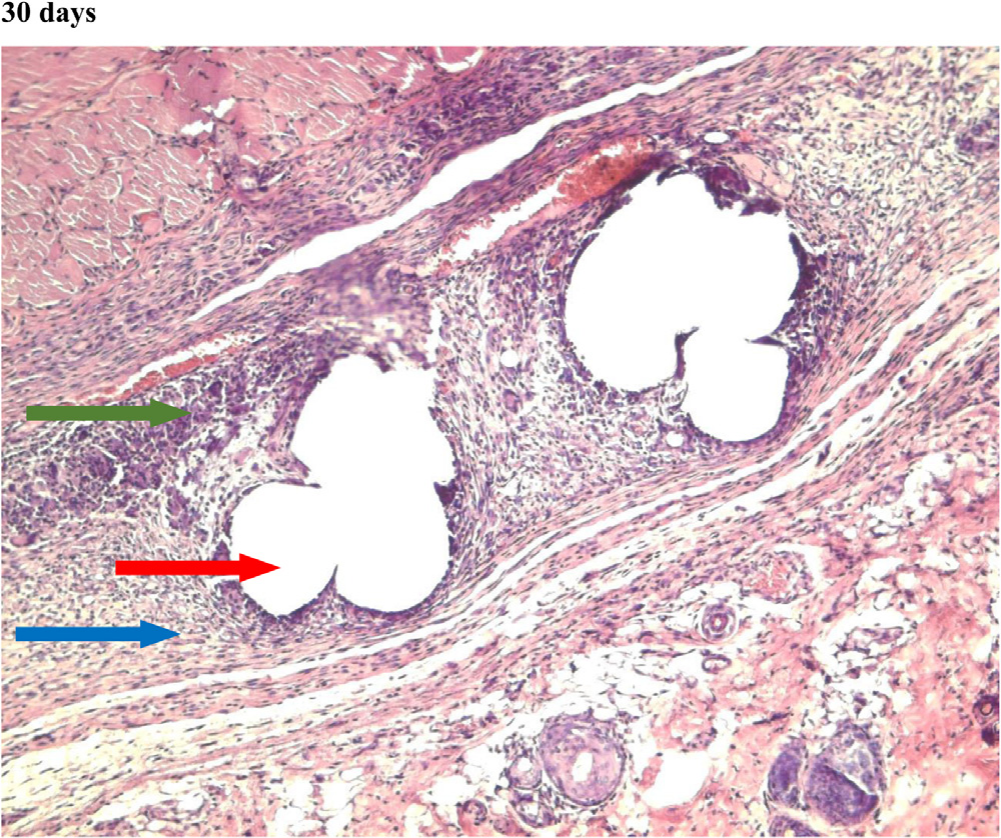
Gr 3-Prolene 30 days.

**Figure 14:**
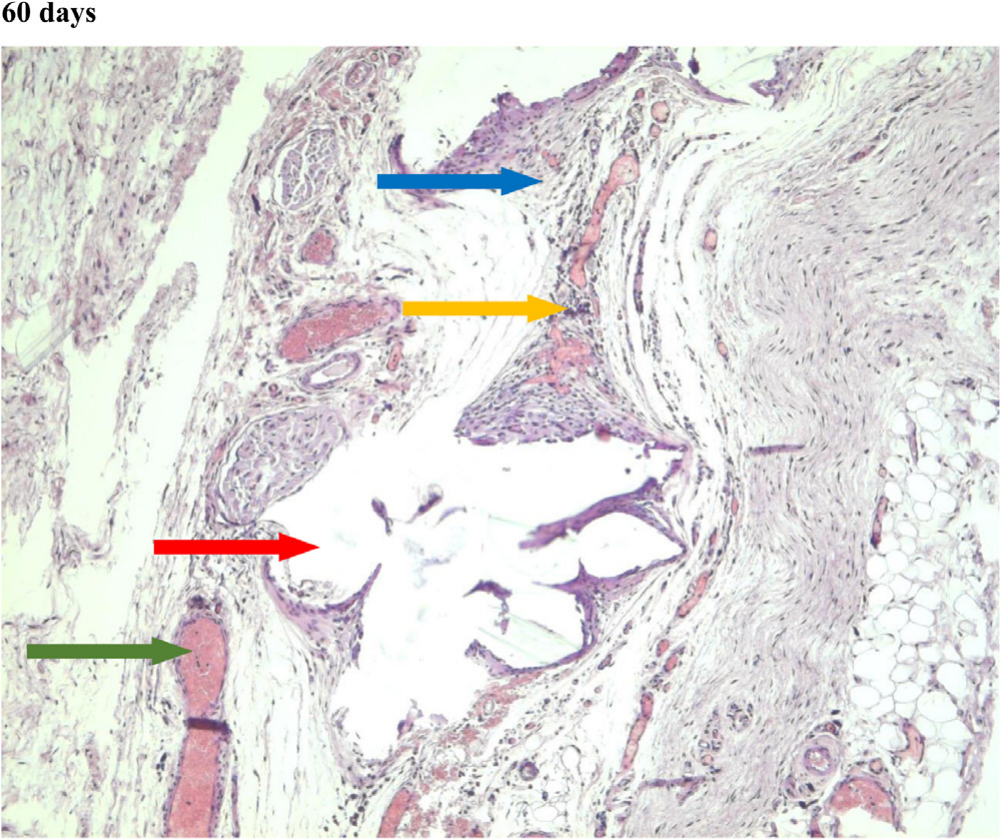
Gr 3-Prolene 60 days.

Statistically significant differences between the study groups at all stages of monitoring were identified. (Table 4)

All statistical data is presented in Tables 2, 3 and 4.

## Discussion

The presented data (in Table 1) indicated that postoperatively during the first week, there was a physiological reaction to the invasive procedure as manifested by a statistically significant difference in the body weight gain of the animals between the groups i.e. the control group and the experimental groups. However, within the experimental groups, there were no statistically significant differences in the weight gain amongst the rodents. On the 14th day of observation, statistically significant differences in the body weight of the mice of the second and third test groups were noted, which, was apparently inferred being due to the continued reaction of the body to the implant. On the 30th day, the lag in the weight gain of the mice were persistent in the second group with partially biodegradable “PARIETENE PROGRIP” implant. On the 60th day, no significant differences in the weight gain among the groups indicated completion of main, energy-dependent biological reactions for implantation of synthetic compositions.

## Conclusion

Histological analysis of titanium mesh implant demonstrated the formation of a firm yet flexible connective tissue meshwork which reduced the possibility of implant contouring and deformation within the thin connective tissues, and thus, it can be considered as a highly suitable implant for static correction in patients with facial paralysis. However, for a more accurate and stable management, postoperatively it was found worth considering a healing timeframe of 30 days for the formation of a full-fledged connective tissue around the grid elements. Thus, the final analysis suggested that the use of titanium mesh for static correction of Facial paralysis and mandibular reconstruction is promising. The results of our clinical application of “Titanium Silk” for static correction of Facial Paralysis – shall be topic of a subsequent article.

Video 1. Stages of the experimental operation.

## Appendix Supplementary materials

Image, video 1
